# A Transcriptomic Analysis of T98G Human Glioblastoma Cells after Exposure to Cadmium-Selenium Quantum Dots Mainly Reveals Alterations in Neuroinflammation Processes and Hypothalamus Regulation

**DOI:** 10.3390/ijms23042267

**Published:** 2022-02-18

**Authors:** Encarnación Fuster, Héctor Candela, Jorge Estévez, Eugenio Vilanova, Miguel A. Sogorb

**Affiliations:** Instituto de Bioingeniería, Universidad Miguel Hernández de Elche, Avenida de la Universidad s/n, 03202 Elche, Spain; e.fuster@umh.es (E.F.); hcandela@umh.es (H.C.); jorge.estevez@umh.es (J.E.); evilanova@umh.es (E.V.)

**Keywords:** quantum dots, nanoparticles, neuroinflammation, gonadotropin-releasing hormone receptor pathway, T98G glioblastoma, nanosafety, in vitro

## Abstract

Quantum dots are nanoparticles with very promising biomedical applications. However, before these applications can be authorized, a complete toxicological assessment of quantum dots toxicity is needed. This work studied the effects of cadmium-selenium quantum dots on the transcriptome of T98G human glioblastoma cells. It was found that 72-h exposure to 40 µg/mL (a dose that reduces cell viability by less than 10%) alters the transcriptome of these cells in biological processes and molecular pathways, which address mainly neuroinflammation and hormonal control of hypothalamus via the gonadotropin-releasing hormone receptor. The biological significance of neuroinflammation alterations is still to be determined because, unlike studies performed with other nanomaterials, the expression of the genes encoding pro-inflammatory interleukins is down-regulated rather than up-regulated. The hormonal control alterations of the hypothalamus pose a new concern about a potential adverse effect of quantum dots on fertility. In any case, more studies are needed to clarify the biological relevance of these findings, and especially to assess the real risk of toxicity derived from quantum dots exposure appearing in physiologically relevant scenarios.

## 1. Introduction

Materials are considered nanomaterials (NMs) when at least one of the dimensions measures less than 100 nm. NMs also possess a large area per unit of volume. Indeed, NMs are defined as those materials with an area/volume ratio over 60 m^2^/cm^3^ [1]. Given this high specific area per unit of volume, NMs display physical characteristics, performance and behavior that differ from the same materials that are not on the nanometric scale. NMs are used across almost all industrial sectors, for example, in the food industry (in packaging, for increasing the nutritional food value or lowering amounts of additives like flavors and colorants), medicine (for targeted drug delivery or in diagnostic tests), in the environment (to more effectively reduce pollution), in cosmetics (colorants, preservatives, UV filters), in pigments, etc.

Quantum dots (QDs) are NMs with unique optical properties [2]. Specifically, QDs possess narrow emission spectra, tuneable broad excitation, high quantum yield and robust photostability. These properties have made conveniently functionalized QDs very promising candidates to be employed in platforms for simultaneous imaging (both in vivo and in vitro), sensing (immunoassay, nucleic acid or single-molecule detection) and therapy (drug delivery) [2,3]. Some authors point out that the future use of QDs for biomedical applications will rely on the minimization of their toxicity [4].

While NMs provide society with economic progress and welfare, they can also pose a human health risk. Therefore, an appropriate risk assessment that warrants adequate risk control before being finally authorized by regulatory agencies is important, as it is for any other application of chemicals that come into contact with humans. The European Union Observatory for Nanomaterials’ Registry [5] shows 322 different NM entries that are already on the European Union market (data consulted on 14 December 2021). However, none of these entries corresponds to QDs. The main aim of this work is to contribute to the study of the neurotoxicity of cadmium-selenium QDs (CdSe-QDs) to facilitate the future authorization of biomedical uses for these promising NMs.

Glial cells play a pivotal role in the maintenance and homeostasis of the central nervous system. The scientific literature reports that metal-containing nanoparticles (NPs) can alter the performance of glia cells by causing inflammation, apoptosis, autophagy, oxidative stress and DNA and mitochondria damage, which lead to neurotoxicity by damaging the blood brain barrier, causing dysfunction of cognition, altering learning and memory and increasing risk of neurodegenerative diseases [6]. Thus, glia cells are relevant for testing the neurotoxicity of CdSe-QDs.

We previously studied the differences between the transcriptome of normal human glial cells and T98G human glioblastoma cells, and found only minimum differences in cellular pathways [7]. Thus no drastic differences in the capacity of these T98G cells’ response to xenobiotics is anticipated, which suggests that they are a good cellular model for testing in vitro toxicity. We previously studied neurotoxicity of silver, titanium dioxide and zinc oxide NPs using this cellular model [7,8]. These T98G cells have previously been used also as model for testing the alterations induced by plant extracts in pro-inflammatory signaling pathways in glial cells [9].

In this work, we explored the in vitro toxicity of CdSe-QDs in T98G cells. We found transcriptomic alterations that addressed mainly the regulation of inflammation and the immune response, the regulation of the gonadotropin-releasing hormone receptor pathway, glial regulation of the developmental process and cell differentiation regulation. 

## 2. Results

### 2.1. Physico-Chemical Properties of CdSe-QDs

**Figure 1:**
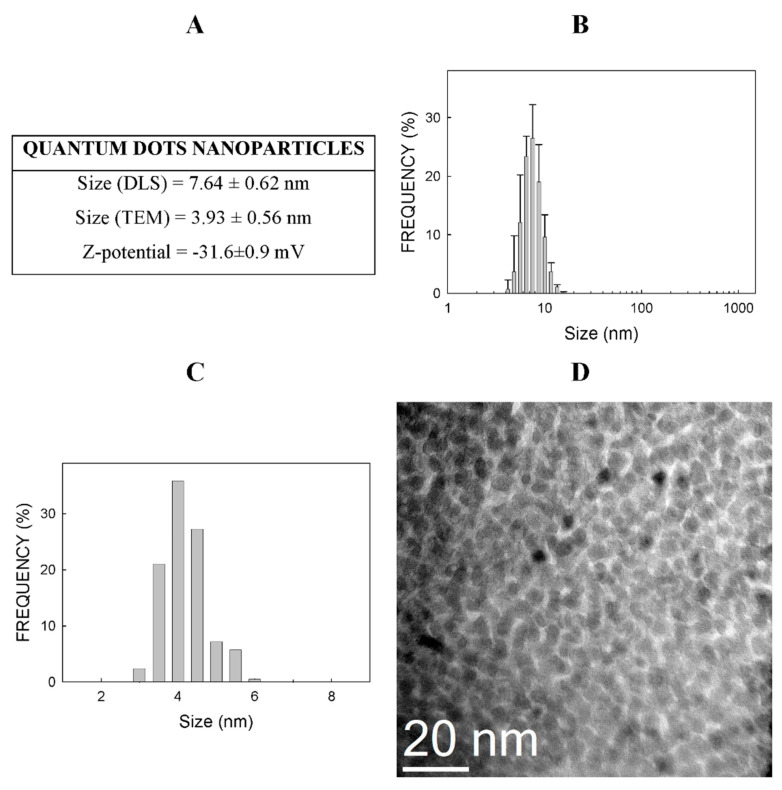
Physico-chemical properties of CdSe-QDs. Physical properties (panel (**A**)) and distribution of sizes as determined by DLS (panel (**B**)) and TEM (panel (**C**)). Panel (**D**) shows a picture of CdSe-QDs under TEM. Determinations were performed by Nanoimmunotech SL (https://nanoimmunotech.eu/en, accessed on 1 February 2022). DLS was analyzed in quintuplicate with a minimum of 10 runs per measurement. The Z-potential was analyzed in triplicate after adjusting the number of runs to each sample’s specific necessity.

Dynamic light scattering (DLS) was used to assess the size and distribution of particles in a 0.5 mg/mL suspension in type I water. The mean the CdSe-QDs’ value was 7.64 ± 0.62 nm, which contained more than 26% of the NPs (Figure 1A). Figure 1B displays the size distribution and shows how 81% of the NPs fell within the 5.6–8.7 nm range.

The Z-potential of these NPs in a 0.5 mg/mL suspension in type I water was −31.6 ± 0.69 mV (Figure 1A). Supplementary Material Figure S1 shows the results of the three independent Z-potential determinations.

Figure 1D is a TEM picture of CdSeQDs. These NPs are spherical with good crystallinity and monodispersity. The mean size determined by transmission electron microscopy (TEM) was 3.93 ± 0.56 nm, which was assumed to be around 36% of all the particles, while about 85% of all the particles fell within the 3.5–4.5 nm range (Figure 1C).

**Figure 2:**
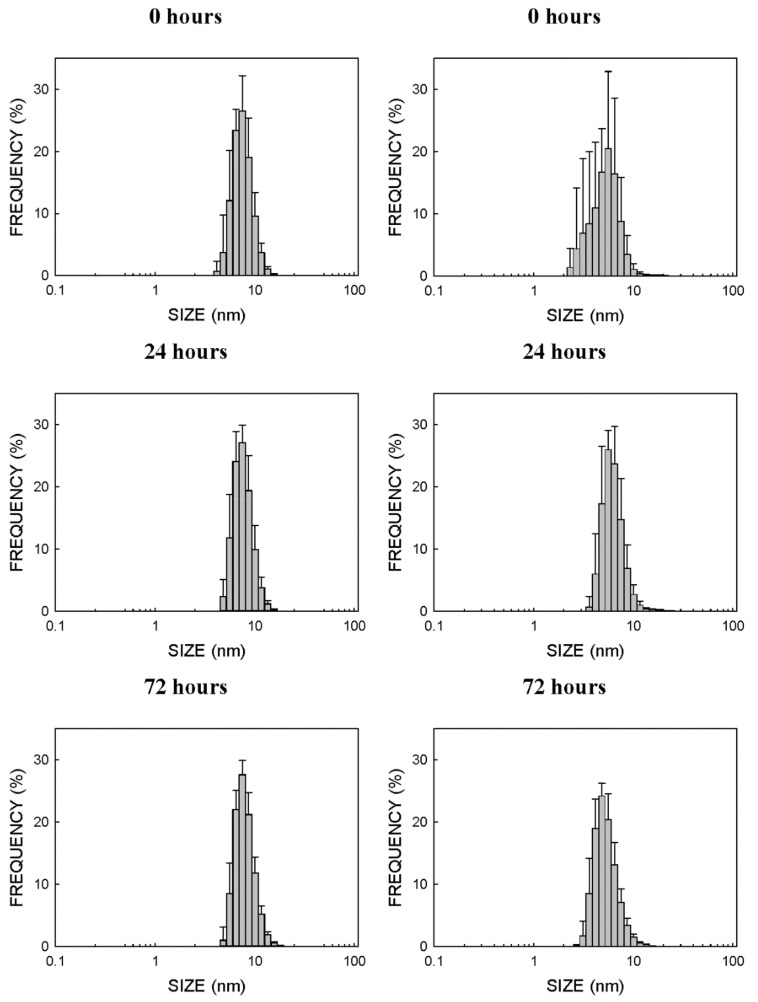
Analysis of the size and distribution of CdSe-QDs in water ((**left**) panels) and cell culture medium ((**right**) panels). Size was determined by DLS. The cell culture medium was the RPMI cellular medium supplemented with 10% FBS. DLS was analyzed in quintuplicate with a minimum of 10 runs per measurement. Determinations were performed by Nanoimmunotech SL (https://nanoimmunotech.eu/en accessed on 1 February 2022)).

Figure 2 shows the plots of the particle sizes determined by DLS for the CdSe-QD suspensions in water and Roswell Park Memorial Institute (RPMI) cell culture medium for up to 72 h. There was no aggregation in either water or the RPMI cell culture medium, and no significant variations in size and distribution were noted. In the water suspension at the three different times, the size with the maximum percentage of NPs (around 27%) was 7.5 nm, and 81%, 82% and 79% of all the NPs were found in a band between 5.6 and 8.7 nm for 0 h, 24 h and 72 h, respectively. Similarly, when CdSe-QDs were suspended in the RPMI cell culture medium supplemented with 10% fetal bovine serum (FBS), the sizes with the largest percentage (between 65% and 77%) of NPs were 5.6 nm (for both after 0 h and 72 h) and 5.0 nm (for 24 h). Most of the NPs (between 81% and 93% of them all) fell within the 3.6–7.5 nm range.

### 2.2. Effects of CdSe-QDs on T98G Cell Viability

**Figure 3:**
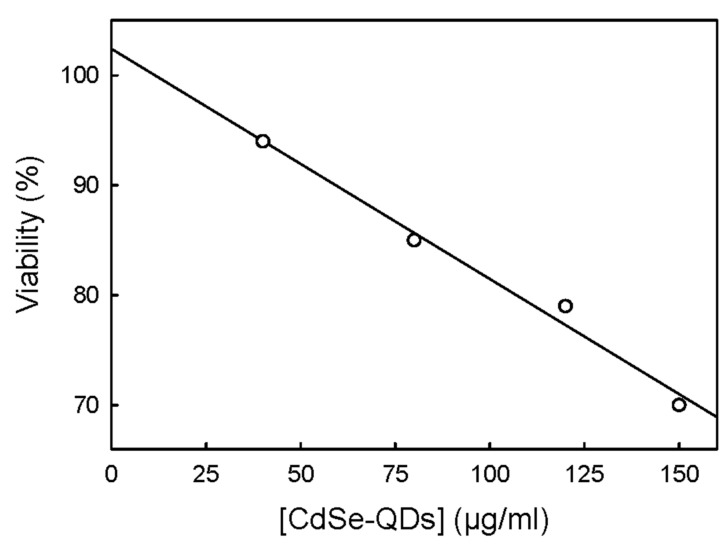
Effect of CdSe-QDs on the cell viability of the T98G human glioblastoma cells. T98G cells were exposed to different CdSe-QDs under the conditions described in Section 4.4. Cell culture viability was determined by the MTT test as described in Section 4.5. Each experimental condition was tested with six different wells of the same cell culture. Another independent experiment with an independent cell culture and six biological replicates per experimental condition yielded similar results. One hundred percent of viability was considered the viability of control (non-exposed) culture.

When T98G cells were exposed to a range of CdSe-QD concentrations between 40 and 150 µg/mL for 72 h cell culture viability lowered in a linear dose-response way (Figure 3). The highest NPs concentration reduced cell culture viability by around 30%. A linear extrapolation of the dose response shown in Figure 3 allowed us to conclude that the concentration of the CdSe-QDs capable of reducing the viability of T98G cells by 50% was 250 µg/mL. This record was 253 µg/mL in another independent experiment with a different culture.

Exposing T98G cells to 30 µg of CdSe-QDs/mL under the conditions described in Section 4.2 reduced viability (determined by the 3-(4,5-dimethylthiazol-2-yl)-2,5-diphenyltetrazoliumbromide (MTT) method) by 9% (Table 1). It was possible to increase the CdSe-QD concentration to 40 µg/mL with no significant reduction in cell viability (lowered by 11%) (Table 1). CdSe-QDs seemed more toxic to mitochondria than to lysosomes because the reduction in viability caused by the exposure to 40 µg/mL of CdSe-QDs was slightly lower (4%) when this endpoint was determined by the neutral red test (Table 1). These data allowed us determine that, under our experimental conditions, the maximum tolerable dose to reduce cell viability by no more than 10% was 40 µg/mL. This concentration was chosen to determine the effect of CdSe-QDs on the transcriptome.

### 2.3. Microscopy

**Figure 4:**
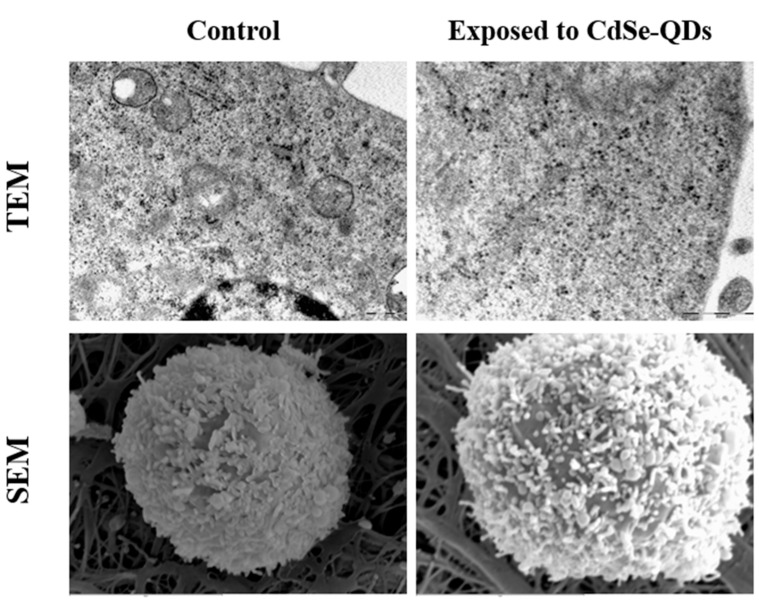
The T98G human glioblastoma cells visualized by TEM and SEM. T98G cells were exposed to 40 µg/mL of CdSe-QDs as described in Section 4.4 and then processed for the electronic microscopy assessment as set out in Section 4.6 according to the performing facility’s internal protocols. A second independent culture yielded similar results. The TEM pictures were obtained both at 37,000×. The SEM pictures were obtained at 8500× (control) and at 8000× (treated).

T98G cells were analyzed after exposure to 40 µg/mL of CdSe-QDs as described in Section 4.6. Neither TEM nor scanning electronic microscopy (SEM) was able to detect the CdSe-QDs bound to the cytoplasmic membrane or incorporated into T98G cells (Figure 4). This result was confirmed in two independent experiments with two different cultures.

### 2.4. Massively Parallel Sequencing of the RNA of the Cells Exposed to CdSe-QDs

#### 2.4.1. RNA Quality

T98G cells were exposed to 40 µg/mL of CdSe-QDs for 72 h following the procedure described in Section 4.2. Afterwards the RNA of cell cultures was extracted and analyzed by the Scientific and Technical Research Area of the University of Murcia (ACTI) with Bioanalyzer 2000 (Agilent). The RNA integrity number (RIN) ranged between 9.6 and 9.8 with a mean ± standard deviation of 9.7 ± 0.1. By way of example, Supplementary Material Figure S2A shows the electropherogram of the second replicate of the T98G cells exposed to NPs. The specific RIN for this sample was 9.8. These samples were shipped under appropriate conditions to MACROGEN Inc. (Seoul, South Korea) to run massive parallel RNA sequencing (RNAseq) experiments. The RIN was determined again in the MACROGEN facilities. The result was 8.0 ± 0.6 (mean ± standard deviation) with a range between 7.0 and 8.7. By way of example, Supplementary Material Figure S2B depicts the electropherogram of the second replicate of the T98G cells exposed to NPs. The specific RIN for this sample was 8.2. A reduction in RNA quality was noted and could be attributed to either degradation during transport or different experimental conditions. Nevertheless, as all the samples had an RIN above the threshold of seven in all cases, RNA was considered apt for use in the RNAseq experiment, which was performed by MACROGEN as Section 4.7 describes.

#### 2.4.2. Alignment of Reads to the Reference Genome

The raw data showed that the control samples had between 30.4 (replicate 2) and 32.0 (replicate 3) million reads, while the number of reads in the samples exposed to CdSe-QDs ranged between 29.8 million reads in replicate 2 and 40.2 million reads in replicate 3 (Table 2). After trimming to delete low-quality and adapter sequences, between 83% and 90% of these reads were kept for further alignment steps to the reference genome (Table 2). This alignment was always higher than 98% for all the replicates of both the treated and control samples (Table 2). Finally, the percentage of duplicated readings fell within the 26.6–31.6% range for replicates 2 and 3 of the treated samples, respectively (Table 2).

#### 2.4.3. Differentially Expressed Genes

In the CdSe-QDs-exposed samples, the bioinformatic analysis identified 544 genes with a statistically different expression from the control samples when the threshold of the false discovery rate (FDR) was set at 5% (Supplementary Material Table S1). The number of repressed genes was 289, while the number of genes with activated expression was 289. Two genes were detected in the exposed cells, but no transcript for these genes was observed in the control cells. These two genes were located in chromosomes 3 and 17, were manually annotated and no function was assigned to them.

The gene with the highest overexpression (log_2_ Fold Change (FC)) = 4.36), and with the transcripts detected in both the control and treated cells, was ENSG00000114857 (Supplementary Material Table S1). This gene encodes the NK-tumor recognition protein, a component of a putative tumor-recognition complex involved in the function of natural killer cells [10]. The gene with the second highest overexpression (log_2_ FC = 2.53) was ENSG00000115353 (Supplementary Material Table S1). This gene encodes a receptor for neuropeptide substance P, which is probably associated with the G proteins that activate a phosphatidylinositol-calcium second messenger system [11].

The gene with the highest under-expression (82%, log_2_ FC = −2.44) was ENSG00000120738 (Supplementary Material Table S1). This gene encodes an early growth response protein, a transcriptional factor involved in the regulation of the cellular response to growth factors, DNA damage and ischemia [12]. ENSG00000170345 was dysregulated in a quite similar extension (78% under expression, log_2_ FC = −2.16) and codes for proto-oncogene c-Fos, a nuclear phosphoprotein that plays an important role in signal transduction, cell proliferation and differentiation by transforming growth factor-beta 1-mediated signaling [13].

**Figure 5:**
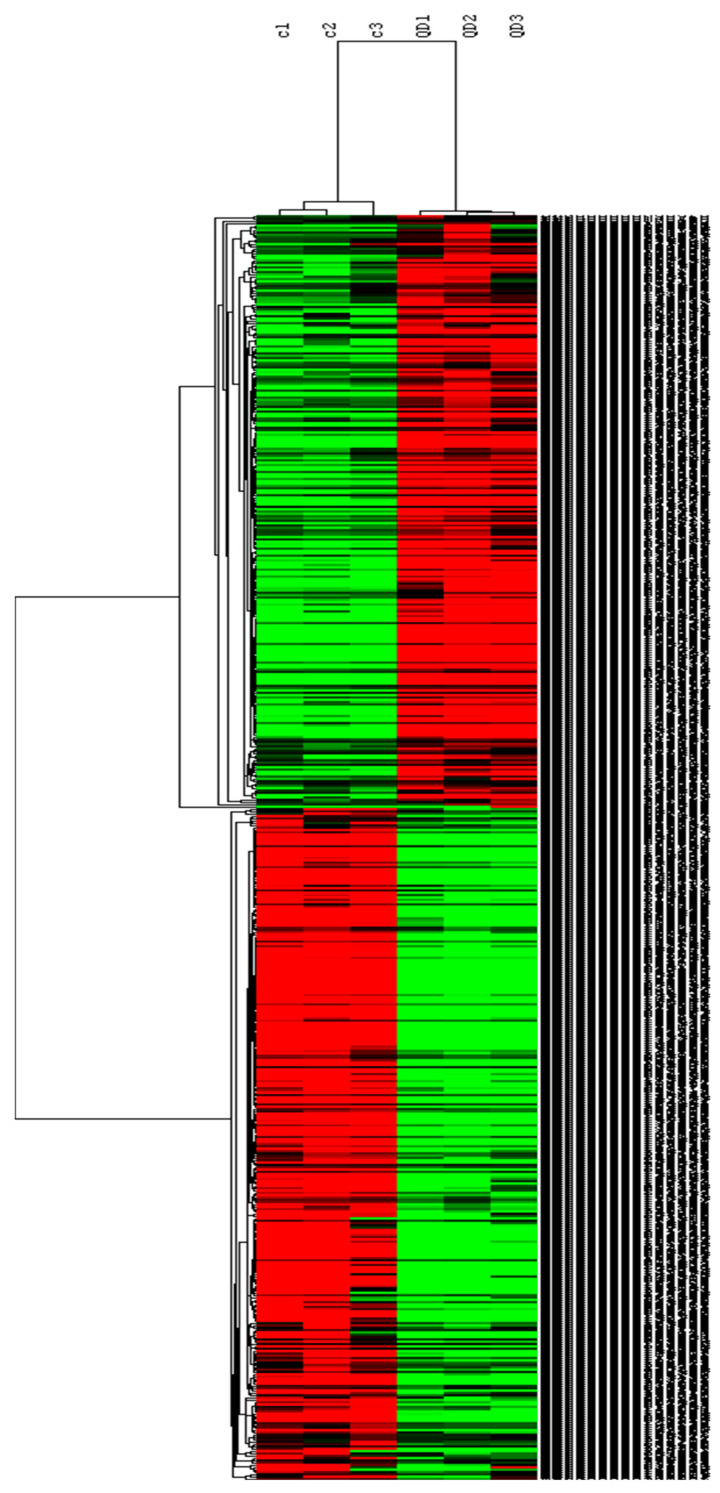
Heat map of the differentially expressed genes in the control samples and the samples treated with CdSe-QDs. The Gene Cluster 3.0 software generated this map by selecting the Hierarchical Grouping option for the studied genes. Green represents under expression, while red annotates overexpression. The whole list of genes shown in this heat map, together with their respective fold changes and levels of statistical significance, are available in Supplementary Material Table S1.

The heat map shown in Figure 5 was prepared to visualize all these genes with an altered expression after CdSe-QD exposure. Dendrograms were obtained by applying a clustering algorithm to samples and differentially expressed genes. As expected, the treated and control samples were clearly distinguished.

#### 2.4.4. Annotation of Differentially Expressed Genes

Supplementary Material Table S1 is a list of the 544 genes whose expression altered in a statistically significant way for an FDR ≤ 0.05. Despite the statistical significance in most cases, the level of dysregulation was moderate. To obtain a clearer picture of the functions altered by CdSe-QD exposure, we decided to perform the Gene Ontology (GO) analysis using only those genes with an FC that equaled or exceeded 2 (log_2_ FC ≥ 1), or those genes with an FC equal to or lower than 0.5 (log_2_ FC ≤ −1). This selection reduced the list of genes to be uploaded to the GO Consortium tool (http://geneontology.org/ accessed on 1 February 2022)) from the 544 initial genes to 59 (31 down-regulated, shown in yellow in Supplementary Material Table S1 and 28 up-regulated, depicted in green in Supplementary Material Table S1).

We determined the overrepresented ontology terms on the list of the 59 genes with the most marked over- or under expression, selected as described above using Fisher’s exact tests, and applying correction with an FDR below 0.05. The list of 11,869 genes expressed in the T98G cells not exposed to CdSe-QDs in the RNAseq experiment was taken as a control. This set of control genes is shown in Supplementary Materials Table S2, and 11,099 of the 11,869 genes were assigned to at least one GO term. The GO Consortium tool unmapped only five genes of the whole set of genes with an altered expression.

Up to six different GO terms were enriched in the complete GO biological process annotation dataset (Table 3). The biological process with the highest enrichment factor (EF) (56) was “*temperature homeostasis*” (GO:0001659), which contained four differentially expressed genes (DEG) on the list. “*Inflammatory response*” (GO:0006954) and “*cytokine-mediated signaling pathway*” (GO:0019221) were biological processes represented in the list of DEG by 8 and 12 genes, which yielded an EF of 7.5 and 6.0, respectively (Table 3). Reactome pathways of GO also found the overrepresentation of DEG in inflammatory response as “*interleukin-10 signaling*” and “*interleukin-4 and interleukin-13 signaling*” which displayed an EF of 36 and 22, respectively (Table 4).

#### 2.4.5. Altered Molecular Pathways

The PANTHER tool yielded 26 altered molecular pathways with the list of the 59 DEG (Table 5). Most of these molecular pathways were altered by a single DEG, while four were altered by two DEG, and four by three DEG or more. These altered molecular pathways meant the marked involvement of the inflammatory processes with altered molecular pathways because of B-cell activation (two DEGs), inflammation mediated by chemokine and cytokine signaling pathways (five DEGs), the interleukin signaling pathway (three DEGs), T-cell activation (one DEG) and also because of participation of genes like interleukin-8, interleukin-6 and iterleukin-1 beta (Table 5). Proto-oncogene c-Fos was the gene involved in most of the altered molecular pathways with a total of eight, followed by transcription factor jun-B and interleukine 8, both with three altered molecular pathways (Table 5). Other molecular pathways with a significant number of genes with altered expression were the gonadotropin-releasing hormone receptor pathway and the CCKR signaling map (both with five DEGs) (Table 5).

## 3. Discussion

This work assessed the effects of CdSe-QDs on the T98G human glioblastoma cells using RNAseq at the maximum non-cytotoxic dose. These NPs altered the transcriptome of T98G cells as regards several molecular pathways, but in a more remarkable way in the pathways involved in neuroinflammation and the control of the hypothalamic-pituitary-gonadal axis via the gonadotropin-releasing hormone receptor pathway. Our approach is based on that proposed by the US National Academy of Sciences [14], which proposes a new paradigm for toxicity testing based firstly on identifying alterations of molecular pathways caused by xenobiotics using preferably human cells, as is the case of the T98G glioblastoma.

### 3.1. Physico-Chemical Properties of Nanoparticles

The Z-potential of the batch of CdSe-QDs used in this work was −31.6 mV and the mean size was 7.6 nm (Figure 1). This Z-potential is similar to that reported for 3-mercaptopropionic acid-modified cadmium telluride QDs of 2.2 and 3.5 nm, which was −31.8 and −26.4 mV, respectively [15]. Similarly, a Z-potential of the same QDs of 4 nm was −12.9 mV [16]. Conversely, a positive Z-potential of polyethylene glycolated CuInS_2_/ZnS QDs (+33.9 mV) has been reported by other authors [17]. Overall, the differences in the Z-potentials of these NPs can be explained based on differences in coating.

CdSe-QDs demonstrated no aggregation tendency, at least not up to 72 h, in both water and cellular medium (Figure 2). This ensured that experiments were performed with a constant particle size. This behavior was not observed for other NPs, such as silver or titanium or zinc oxides, which demonstrated an aggregation tendency and, therefore, an increased mean particle size over time with a more marked tendency in water than in cellular medium [7,8].

### 3.2. Cytotoxicity

The batch of CdSe-QDs used in this work exhibited relatively low cytotoxicity, with an estimated concentration capable of reducing viability by 50% of around 250 µg/mL (Figure 3) and a maximum tolerable dose of 40 µg/mL (Table 1) after 72 h of exposure. However, Huang and Tang [18] reviewed the cytotoxicity of different cadmium-based QDs. They reported the need to apply doses between 20 and 160 µg/mL and exposures ranging from 4 h to 24 h for a 50% reduction in viability with a range of cellular lines. He and co-workers [19] reported that the cytotoxicity of QDs was closely related to cadmium concentration. This suggests that our batch of CdSe-QDs contains a relatively small amount of cadmium. Indeed the cytotoxicity records found in our work are similar to the 250 µg/mL of a QD preparation lacking cadmium that led to 50% cell viability reductions in PC12 cells after 48 h of exposure [17].

Turovsky and co-workers [20] reported that selenium nanoparticles are able to protect cortical astrocytes and neurons against ischemic brain injuries inhibiting apoptosis inactivating caspase 3 and recruiting nuclear factors Nrf2 and SOCS3/STAT3. We hypostatize that these mechanisms could be protecting T98G cells against the deleterious effects of the cadmium contained in our CdSe-QDs, which could be also an alternative explanation for the low cytotoxicity.

### 3.3. Cellular Uptake

CdSe-QDs were not incorporated into T98G cells or remained bound to the cytoplasmic membrane under our experimental conditions (Figure 4). This behavior is similar to the no uptake of silver NPs being noted in T98G cells [7], but is contrary to the uptake of titanium dioxide NPs in these same cells [8]. Other QDs NPs, such as CdTe-QDs coated with 3-mercaptopropionic acid can be deposited in the motor neurons of the nematode *Caenorhabditis elegans* [16]. Similarly, polyethylene glycolated CuInS_2_/ZnS QDs also incorporated into PC12 cells in a concentration-dependent way [17]. These two different QDs were incorporated into cells own very different Z-potentials (−12.9 vs + 33.9 mV). Thus, it would seem that neither the Z-potential nor the NP coating are responsible for incorporation into cells. Our CdSe-QD batch was also functionalized with a negatively charged coating similarly to the 3-mercaptopropionicacid CdTe-QDs, but did not incorporate into T98G cells, which suggests that these differences might be due to methodological procedures. Despite lack of incorporation, CdSe-QDs had notable effects on the transcriptome of T98G cells as the expression of 544 genes was statistically altered (Supplementary Material Table S1).

### 3.4. Altered Molecular Pathways and Biological Processes

#### 3.4.1. Gonadotropin-Releasing Hormone Receptor Pathway

Table 5 provides a number of dysregulated pathways after exposure to CdSe-QDs. One of these dysregulated pathways is the gonadotropin-releasing hormone receptor pathway, which shows down-regulation in four of its genes and up-regulation in a fifth one. Gonadotropin-releasing hormone determines sexual development and adult reproductive function, and glia is greatly involved in the regulation of gonadotropin-releasing hormone neurons via the excretion of bioactive molecules, which exert paracrine effects, and also through glia-neuron interactions by adhesive molecules [21]. The disruption of the hypothalamic-pituitary-gonadal axis controlled by the gonadotropin-releasing hormone leads to a condition known as hypogonadotropic hypogonadism, which impairs reproductive function [22].

The alteration of the gonadotropin-releasing hormone receptor pathway is also supported by the overrepresentation of the biological processes *“positive regulation of developmental process*” (GO:0051094) and “*regulation of cell differentiation*” (GO:0045595) in the set of DEG (Table 3). Indeed glia plays a pivotal role in neural maturation of the circuits controlling gonadotropin-releasing hormone secretion and in regulating neuronal connectivity in the brain regions involved in the control of reproduction [23].

The biological process with the highest EF (56) is “*temperature homeostasis*” (GO:0001659) (Table 3). Core body temperature is regulated by hypothalamus via hormonal mechanisms [24]. In this way, the aforementioned alterations to gonadotropin regulation may also influence the reported alteration to temperature homeostasis.

We were unable to find references to the potential reproductive toxicity of QDs in the open scientific literature. However, the information provided in this work about alterations to the regulation of hypothalamus via variations in the gonadotropin-releasing hormone receptor pathway indicates the need to explore and assess potential reproductive toxicity caused by exposure to QDs.

#### 3.4.2. Inflammation and Immune System Alterations

The exposure of T98G cells to CdSe-QDs brought about the statistically significant alterations to the expression of the genes involved in a number of molecular pathways (Table 5), biological processes (Table 3) and reactome pathways (Table 4). The biological relevance of these alterations still has to be determined in some cases, but there are a number of issues that address the regulation of the immune and inflammatory systems, specifically, B-cell activation, inflammation mediated by chemokine and cytokine signaling pathways, interleukin signaling pathway, T-cell activation, “*inflammatory response*” (GO:0006954), “*cytokine-mediated signaling pathway*” (GO:0019221), “*interleukin 10 signaling*” and “*interleukin 4 and 13 signaling*”. These results are similar to those previously reported in these same T98G cells with titanium dioxide [8] and silver [7], in the rat hippocampus with CdTe-QD NPs [15] and in human hepatic cell line L02 with CdSe/ZnS-QD NPs [25]. Another in vivo study with mice also demonstrates that CdTe/ZnS QDs can polarize microglia in the brain and cause secondary inflammatory damage to neurons [26]. Moreover, inflammation of neurons and glia is acknowledged as a potential mechanism of neurotoxicity induced by metal containing QDs [6,27,28].

Inflammation is a protective mechanism against external deleterious stimuli, such as virus, bacterial infections or xenobiotic insults. However, the exacerbation of inflammation can elicit tissue injury when not controlled; for example, neuroinflammation is postulated as one of the key events needed to trigger parkinsonian motor deficits [29]. However, the physiological connotations of the alterations to inflammatory and immune response shown in Table 3, Table 4 and Table 5 are still unclear as it is noted that the expression of pro-inflammatory interleukins 6, 8 and 32 respectively lowers by 72%, 69% and 45%, while the expression of interleukin 1 beta increases by 298% (Supplementary Material Table S1). The reduced expression of the pro-inflammatory cytokines induced by CdSe-QDs falls in line with the findings of Tosic and co-workers [30], who noted that graphene-QDs suppress experimental autoimmune encephalomyelitis in rats by reducing spinal cord demyelination and axonal damage, and T-helper 1 development and spinal cord inflammation, and by blocking (in vitro) T-cell-induced oligodendrocyte/neuron damage. A recent study reports that Se-QDs are able to alleviate neuroinflammation and to improve memory impairment, learning and memory ability in a mouse model of Alzheimer’s disease [31]. Overall, more studies are needed to determine whether the reported alterations to immune and inflammatory pathways and processes are able to induce the adverse typically described effects or, conversely, can offer the potential beneficial effects described by Tosic et al. [30] and by Guo et al. [31].

### 3.5. Risk

The results herein presented identify several potential hazards for CdSe-QDs. However, these hazards have been identified in vitro at relatively high concentrations and these concentrations are not expected to be physiologically relevant for in vivo exposures. Thus more research is needed to assess the real risk of these hazards during biologically relevant exposures and to determine whether the reported reductions in the expression of pro-inflammatory interleukins can imply a potential therapeutic application for treating inflammatory-mediated diseases.

## 4. Materials and Methods

### 4.1. Nanoparticles

The NPs of carboxylic acid functionalized CdSe-QDs (catalogue no. QD-1, batch 17001) were purchased from SCHARLAB SL, (Barcelona, Spain) (www.scharlab.com accessed on 1 February 2022)). CdSe-QDs were provided in a sterile milli-Q water suspension at a concentration of 1 mg/mL. This suspension was sonicated at 110 W for 10 min before preparing a stock suspension of 400 µg/mL in phosphate buffer saline, which was stored at 4 °C in the dark until it was used.

For the experiments, CdSe-QD suspensions in cell culture medium were prepared at the appropriate concentration immediately before cellular exposures began. For this purpose, the 400 µg/mL stock suspension was sonicated again for 10 min at 110 W before adding the required aliquot to the appropriate cellular media volume.

### 4.2. Physico-Chemical Characterisation of QD-NPs

The NP supplier provided the TEM image and the ultraviolet-visible absorption spectra shown in the Supplementary Material (Figure S3). The NPs used in this work are spherical (Supplementary Material Figure S3A) with a peak of absorption at around 570 nm (Supplementary Material Figure S3B). We requested an additional physical characterization of the CdSe-QDs from Nanoimmunotech SL (Madrid, Spain) (https://nanoimmunotech.eu/en accessed on 1 February 2022)). This characterization included determinations of particle size, Z-potential, TEM and stability in cell culture medium.

Size, which was determined by DLS, and Z-potential were assessed at 25 °C in type 1 water (18 MΩ.cm). DLS was analyzed in quintuplicate with a minimum of 10 runs per measurement. The Z-potential was analyzed in triplicate after adjusting the number of runs to each sample’s specific necessity. The samples for TEM were allocated in copper grids with carbon film, which were air-dried before taking images under a TECNAI F30 (300 kV) microscope. To study stability, the NPs suspensions of CdSe-QDs were prepared in RPMI medium supplemented with 10% FBS or sterile milli-Q water type 1. These suspensions were incubated at 37 °C for 0 h, 24 h and 72 h before determining size by DLS.

### 4.3. Cellular Cultures

The T98G human glioblastoma cells were purchased from the European Collection of Authenticated Cell Cultures (UK) (catalogue no. 92090213) (https://www.phe-culturecollections.org.uk/collections/ecacc.aspx accessed on 1 February 2022). These cells derived from a glioblastoma multiform tumor from a 61-year-old Caucasian male and are a polyploid variant of T98 cells. T98G cells exhibit transformed immortality, but are arrested in the G1 phase under stationary phase conditions [32].

Cells were cultured at 37 °C in a 5% CO_2_ atmosphere as previously described [7,8]. Briefly, cells were grown and expanded until confluence using P100 TPP plates in Dulbecco’s modified Eagle Medium-GlutaMAX™-I with glucose and sodium pyruvate supplemented with FBS and non-essential amino acids, penicillin and streptomycin. Once cells had reached confluence in P100 TPP plates, they were trypsinized and subcultured at the appropriate density according to the specific experiment in either P60 TPP plates or 96-well trays.

### 4.4. Cellular Exposure to CdSe-QDs

Cells were seeded on trays in complete medium on day 0. On day 1, the medium was removed and fresh medium containing CdSe-QDs at appropriate concentrations was added for 72 h. This exposure time was chosen as representative of at least a sub-chronic exposure for these cells.

### 4.5. Cellular Viability Tests

The effects of CdSe-QDs on T98G human glioblastoma cell viability were determined by two different cytotoxicity tests: The MTT test, which targets toxicity on mitochondria; and the neutral red uptake test, which targets toxicity on the lysosome. For both tests, each experimental condition was assayed during all the independent experiments with six biological replicates. In all cases, the percentage of viability of the cells exposed to CdSe-QDs was estimated by assuming the absorbance of the control cultures (not exposed to NPs) as 100% viability. In all the experiments, cytotoxicity-positive controls were run using 10 µg/mL of CuSO_4_, which was able to reduce the viability of the exposed cultures by around 80%.

T98G cells were seeded in 96-well plates at 10,000 cells/plate and 5000 cells/plate on day 0 for the neutral red test and the MTT test, respectively. After the 72-h exposure to CdSe-QDs as described in Section 4.4, cytotoxicity was assayed in line with Fuster et al. [7,8]. Red neutral test was assayed using the kit provided by BioVision Inc. (product number K 447) as recommended by the supplier. For the MTT test, cells were incubated for 3 h with MTT in the dark before lysis with dimethyl sulfoxide and absorbance determination.

### 4.6. Electronic Microscopy

Microscopy services were provided by ACTI (https://www.um.es/web/acti/ accessed on 1 February 2022) (University of Murcia, Murcia, Spain). T98G cells (300,000 cells/plate) were exposed to 40 µg/mL of CdSe-QDs as described in Section 4.4. Next, NPs were removed, and cells were washed with phosphate buffer saline and trypsinized for detachment. Aliquots of 100,000 cells were pelleted and resuspended with fixative solution (3% glutaraldehyde in 0.1 M cacodylate buffer, pH 7.4) and were left 5 min at room temperature, followed by 1 h at 4 °C before removing the fixative solution and resuspending cells in 8% sucrose in 0.1 M cacodylate buffer, pH 7.4. Cells were then transported to the ACTI facilities in an ice bath, where they were treated for TEM and SEM according to previously described internal procedures [7].

### 4.7. Massively Parallel RNA Sequencing

T98G cells were seeded at 160,000 cells/P60 TPP plate on day 0 and exposed for 72 h to 40 µg of CdSe-QDs/mL as described previously in Section 2.4. This CdSe-QD concentration was chosen as the maximum concentration capable of lowering cell viability by no more than 10% (see the Results section about the cell viability tests for details). The control samples were cultured in parallel without NPs. Each experimental condition was run in triplicate. Immediately after exposure finished, RNA was isolated with Trizol reagent following previously described standard procedures (Fuster et al., 2020). The RNA integrity number (RIN) was determined at the ACTI facilities using Bioanalyzer 2000 (Agilent). Only those samples with an RIN over seven were considered apt for the RNAseq experiments.

RNAseq was performed by MACROGEN Inc. (https://dna.macrogen.com/eng/ accessed on 1 Febryary 2022) with the Illumina platform using paired-end 101-bp reads. The RNAseq raw data are deposited in the NCBI Sequence Read Archive (SRA) (https://www.ncbi.nlm.nih.gov/sra/ accessed on 1 February 2022) with accession number SAMN13151876.

### 4.8. Bioinformatic Analysis

The following workflow was followed for the bioinformatic analysis. Firstly, the low-quality and remaining adapter sequences present in reads were trimmed with Trimmomatic v. 0.36. Then reads were mapped to the GRCh38 version of the human reference genome with Hisat2 v. 2.1.0. The resulting SAM files were transformed into BAM files with Samtools v. 0.1.19. Finally, the gene expression level was quantified with Cufflinks v. 2.2.1, and statistical comparisons were made with the same software. Details of the analysis are published in Fuster et al. [7]. Cluster v. 3.0 [33] was used to prepare heat map, which were further visualized with Java Tree View software [34].

### 4.9. Ontological Analysis

The GO (http://www.geneontology.org/ accessed on 1 February 2022) and Protein ANalysis THrough Evolutionary Relationships (PANTHER) (http://pantherdb.org/ accessed on 1 February 2022) resources were used to assign ontology to the differentially expressed genes and to determine the biological pathways in which they were involved.

## 5. Conclusions

In the present work, we have demonstrated that CdSe-QD NPs are able to alter transcriptome of T98G human cell glioblastoma by inducing changes in a number of biological pathways. Among these altered biological pathways, those with highest relevance are the alterations in the gonadotropin-releasing hormone receptor pathway and the alterations in the inflammation. The first one suggests that these NPs could alter reproductive performance via hormonal disturbance in the hypothalamus, and the second one is still of unknown consequences on neuroinflammation since the expressions of most of the altered proinflammatory interleukins were downregulated rather than upregulated.

## Figures and Tables

**Table 1:** Effect of CdSe-QDs on the cell viability of the T98G human glioblastoma cells. The T98G human glioblastoma cells were exposed to NPs, as defined in Section 4.4 (Materials and Methods). Next, NPs were removed, and culture viability was determined by either the MTT or neutral red tests. The mean ± standard deviation of the percentage of viability for (n) independent experiments (run with independent cell cultures) is displayed. Each independent experiment was performed with six wells of the same cell culture. Each experiment was run simultaneously with a positive control of CuSO_4_ that reduced cell viability by around 80%.

Concentration (µg/mL)	Test	% of Viability (n)
40	MTT	89 ± 8 (9)
30	MTT	91 ± 1 (3)
40	Neutral red	96 ± 1 (7)

**Table 2:** Alignment of the sequences to the reference genome. This experiment was run with CdSe-QDs in parallel with the experiments with titanium dioxide and zinc oxide NPs and, therefore, shares the controls with the data published by Fuster and coworkers [8].

	Control Samples			Treated Samples		
	Replicate 1	Replicate 2	Replicate 3	Replicate 1	Replicate 2	Replicate 3
Initial	31,016,624	30,419,346	32,022,288	34,786,612	29,827,860	40,211,724
Filtered	26,681,128	26,192,232	26,451,976	29,101,028	24,773,250	36,251,964
Aligned (%)	26,342,795 (98.7)	25,793,274 (98.5)	26,083,336 (98.6)	28,717,867 (98.7)	24,455,042 (98.7)	35,782,526 (98.7)
Duplicated (%)	28.1	28.1	26.9	28.7	26.6	31.6

**Table 3:** The biological process ontology terms overrepresented in the differentially expressed genes in the T98G human glioblastoma cells exposed for 72 h to 40 µg/mL of CdSe-QDs. Only the results for FDR *p* < 0.05 are displayed. DEG = Differentially Expressed Genes; EF = Enrichment factor; FDR = False Discovery Rate.

Biological Process Sub-Ontology (GO Term)	Genes in Reference List	Genes among DEG	Expected among DEG	EF	*p* Value	FDR
Temperature homeostasis (GO:0001659)	14	4	0.07	56	1.78 × 10^−6^	8.35 × 10^−3^
Inflammatory response (GO:0006954)	208	8	1.07	7.5	1.27 × 10^−5^	2.24 × 10^−2^
Cytokine-mediated signaling pathway (GO:0019221)	390	12	2.00	6.0	6.66 × 10^−7^	9.38 × 10^−3^
Positive regulation of developmental process (GO:0051094)	746	15	3.83	3.9	4.18 × 10^−6^	1.18 × 10^−2^
Regulation of cell differentiation (GO:0045595)	966	18	4.96	3.63	1.01 × 10^−6^	7.13 × 10^−3^
Regulation of cell death (GO:0010941)	1091	17	5.61	3.0	2.36 × 10^−5^	3.32 × 10^−2^

**Table 4:** The reactome pathway ontology terms overrepresented in the differentially expressed genes in the T98G human glioblastoma cells exposed for 72 h to 40 µg/mL of CdSe-QDs. Only the results for FDR *p* < 0.05 are displayed. DEG = Differentially Expressed Genes; EF = Enrichment factor; FDR = False Discovery Rate.

Reactome Pathway	Genes in Reference List	Genes among DEG	Expected among DEG	EF	*p* Value	FDR
Interleukin-10 signaling	16	3	0.08	36	1.16 × 10^−4^	4.92 × 10^−2^
Interleukin-4 and Interleukin-13 signaling	62	7	0.32	22	5.27 × 10^−8^	1.12 × 10^−4^
Class A/1 (Rhodopsin-like receptors)	50	5	0.26	19	8.36 × 10^−6^	8.87 × 10^−3^

**Table 5:** Molecular pathways altered in the T98G human glioblastoma exposed for 72 h to 40 µg/mL of CdSe-QDs.

Pathway	Genes Involved	Log_2_ Fold Change
5-Hydroxytryptamine degradation	Aldehyde dehydrogenase family 1 member A3	1.12
Angiogenesis	Proto-oncogene c-fos	−2.16
	Tissue factor F3 ortholog	−1.05
Angiotensin II-stimulated signaling through G proteins and beta-arrestin	Early growth response protein 1	−2.44
Apoptosis signaling pathway	Proto-oncogene c-Fos	−2.16
	Baculoviral IAP repeat-containing protein 3	−1.19
B cell activation	Proto-oncogene c-Fos	−2.16
	B-cell receptor CD22	1.41
Blood coagulation	Tissue factor F3	−1.04
CCKR signaling map	Early growth response protein 1	−2.44
	Proto-oncogene c-Fos	−2.16
	Interleukin-8	−1.67
	Substance-P receptor	2.54
	Baculoviral IAP repeat-containing protein 3	−1.19
Coenzyme A biosynthesis	Pantothenate kinase 1	1.04
Dopamine receptor-mediated signaling pathway	D(2) dopamine receptor	1.07
EGF receptor signaling pathway	Protein sprouty homolog 4	−1.98
FGF signaling pathway	Protein sprouty homolog 4	−1.98
Gonadotropin-releasing hormone receptor pathway	Early growth response protein 1	−2.44
	Proto-oncogene c-Fos	−2.16
	D(2) dopamine receptor	1.07
	DNA-binding protein inhibitor ID-3	−1.07
	Transcription factor jun-B	−1.16
Heterotrimeric G-protein signaling pathway-Gi alpha and Gs alpha-mediated pathway	D(2) dopamine receptor	1.07
Heterotrimeric G-protein signaling pathway-Gq alpha and Go alpha-mediated pathway	D(2) dopamine receptor	1.07
	B2 bradykinin receptor	1.06
Huntington disease	Proto-oncogene c-Fos	−2.16
Inflammation mediated by chemokine and cytokine signaling pathway	Interleukin-8	−1.67
	Transcription factor jun-B	−1.16
	Integrin alpha-M	−1.80
	Interleukin-6	−1.84
	Interleukin-1 beta	1.57
Insulin/IGF pathway-mitogen activated protein kinase kinase/MAP kinase cascade	Proto-oncogene c-Fos	−2.16
Integrin signaling pathway	Integrin alpha-M	−1.80
Interleukin signaling pathway	Proto-oncogene c-Fos	−2.16
	Interleukin-8	−1.67
	Interleukin-6	−1.84
Nicotine pharmacodynamics pathway	D(2) dopamine receptor	1.07
PDGF signaling pathway	Proto-oncogene c-Fos	−2.16
T cell activation	Proto-oncogene c-Fos	−2.16
TGF-beta signaling pathway	Transcription factor jun-B	−1.16

## Data Availability

The RNAseq data presented in this study are openly available in Sequence Read Archive (SRA) (https://www.ncbi.nlm.nih.gov/sra/, accessed on February 2022) with Bio Project accession number PRJNA580150.

## Supplementary Materials

The following are available online at https://www.mdpi.com/article/10.3390/ijms23042267/s1.
