# Supplementary material for: A Transcriptomic Analysis of T98G Human Glioblastoma Cells after Exposure to Cadmium-Selenium Quantum Dots Mainly Reveals Alterations in Neuroinflammation Processes and Hypothalamus Regulation
Source: Int J Mol Sci. 2022 Feb 18;23(4):2267. doi: 10.3390/ijms23042267 (PMC8877384; doi:10.3390/ijms23042267)
Supplement: Supplementary file 1 [file ijms-23-02267-s001.zip › ijms-1597766-supplementary.pdf]

International Journal of
Molecular Sciences

MDPI

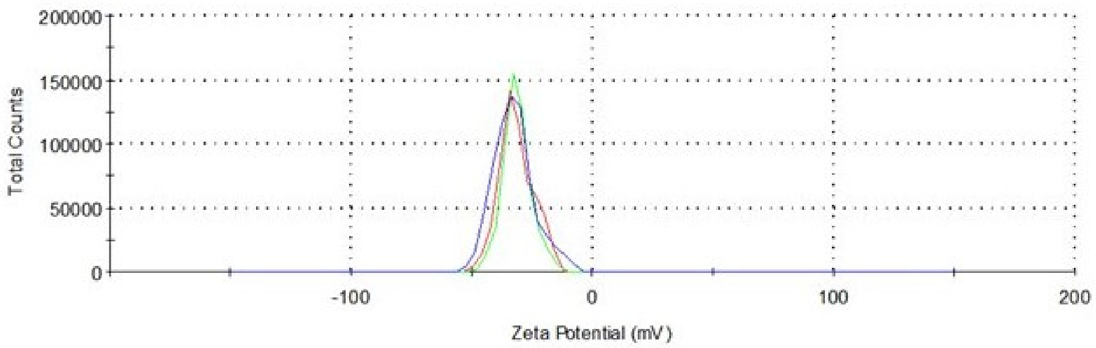

Figure S1. Analysis of the Z-potential for CdSe-QDs in type I water. Determinations were performed by Nanoimmunotech SL (https://nanoimmunotech.eu/en).

A

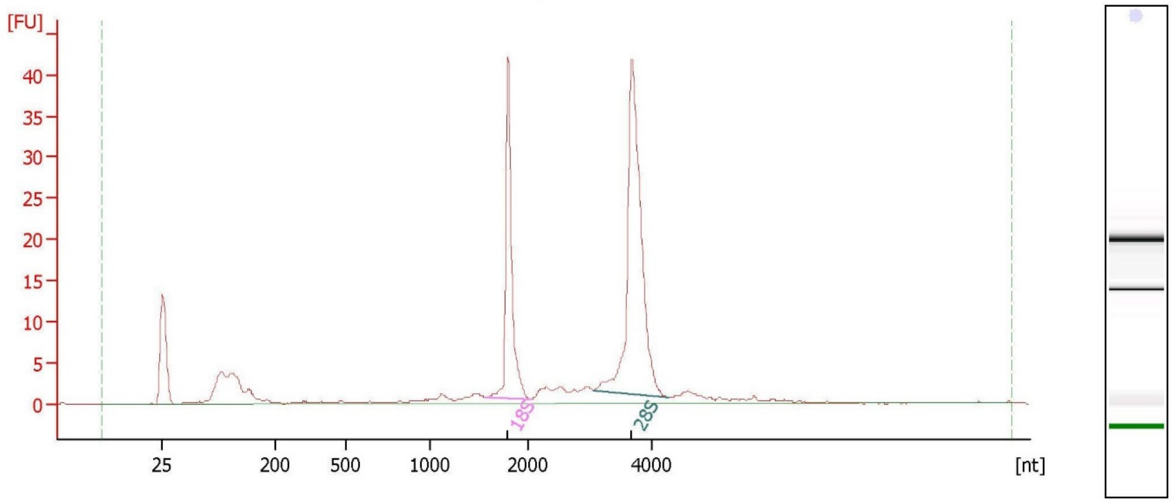

B

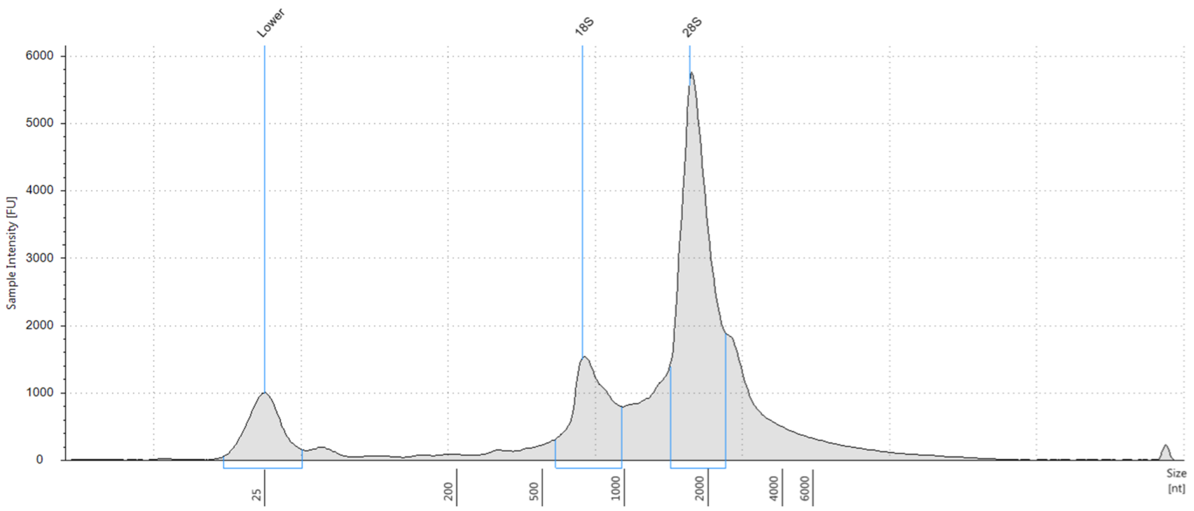

Figure S2. Examples of the electropherograms used to estimate the RIN in the samples used for RNAseq. The picture in panel (**A**) corresponds to the RNA of replicate 2 of the cells exposed to 40 µg/mL of the CdSe-QDs obtained immediately after RNA isolation, and corresponds to a RIN of 9.8. The picture in panel (**B**) corresponds to the same sample analyzed by MACROGEN Inc. after being transported to South Korea and corresponds to an RIN of 8.2.

A **B**

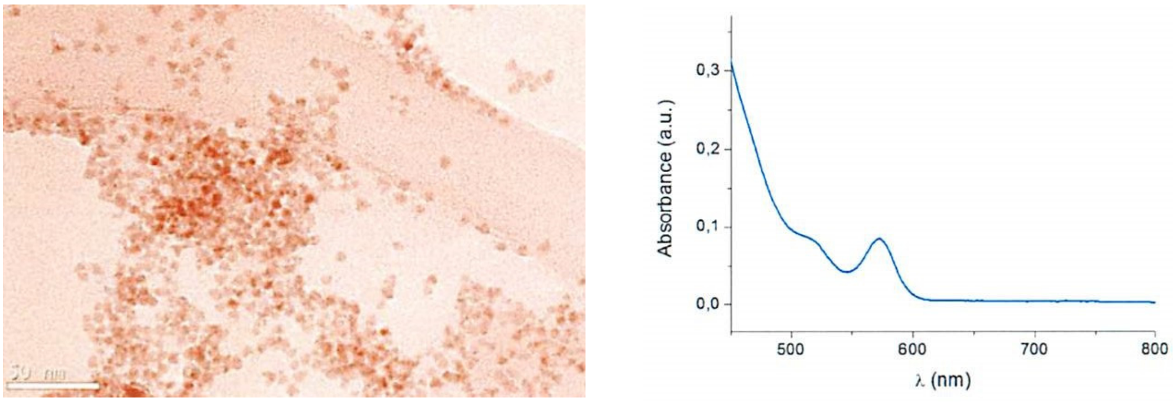

Figure S3. Preliminary physical characterization of the CdSe-QD NPs. TEM image (**A**) and the ultraviolet-visible absorption spectrum of the CdSe-QDs (**B**) used in this work. The company providing the NPs batch supplied the information.
